# Supplementary material for: Overexpression of bmp4, dazl, nanos3 and sycp2 in Hu Sheep Leydig Cells Using CRISPR/dcas9 System Promoted Male Germ Cell Related Gene Expression
Source: Biology (Basel). 2022 Feb 11;11(2):289. doi: 10.3390/biology11020289 (PMC8869737; doi:10.3390/biology11020289)
Supplement: Supplementary file 1 [file biology-11-00289-s001.zip › Figure S1.pdf]

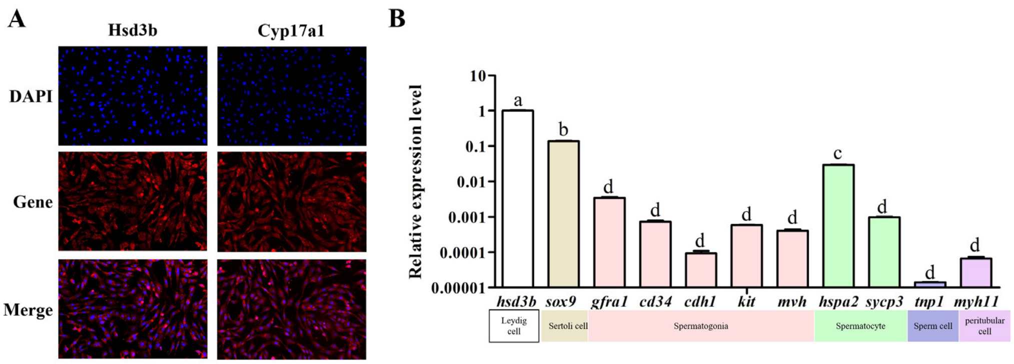

Figure S1. Identification of isolated sheep Leydig cells. A Immunofluorescence of marker gene Hsd3b and Cyp17a1 was analyzed the purity of the isolated sheep leydig cells. DAPI was indicated the nuclear staining, and the results was photographed under laser scanning confocal microscope at a magnification of 40×. B Expression levels of marker genes in different testis cell types.
