# Supplementary figures and images for: Overexpression of bmp4, dazl, nanos3 and sycp2 in Hu Sheep Leydig Cells Using CRISPR/dcas9 System Promoted Male Germ Cell Related Gene Expression
Source: Biology (Basel). 2022 Feb 11;11(2):289. doi: 10.3390/biology11020289 (PMC8869737; doi:10.3390/biology11020289)

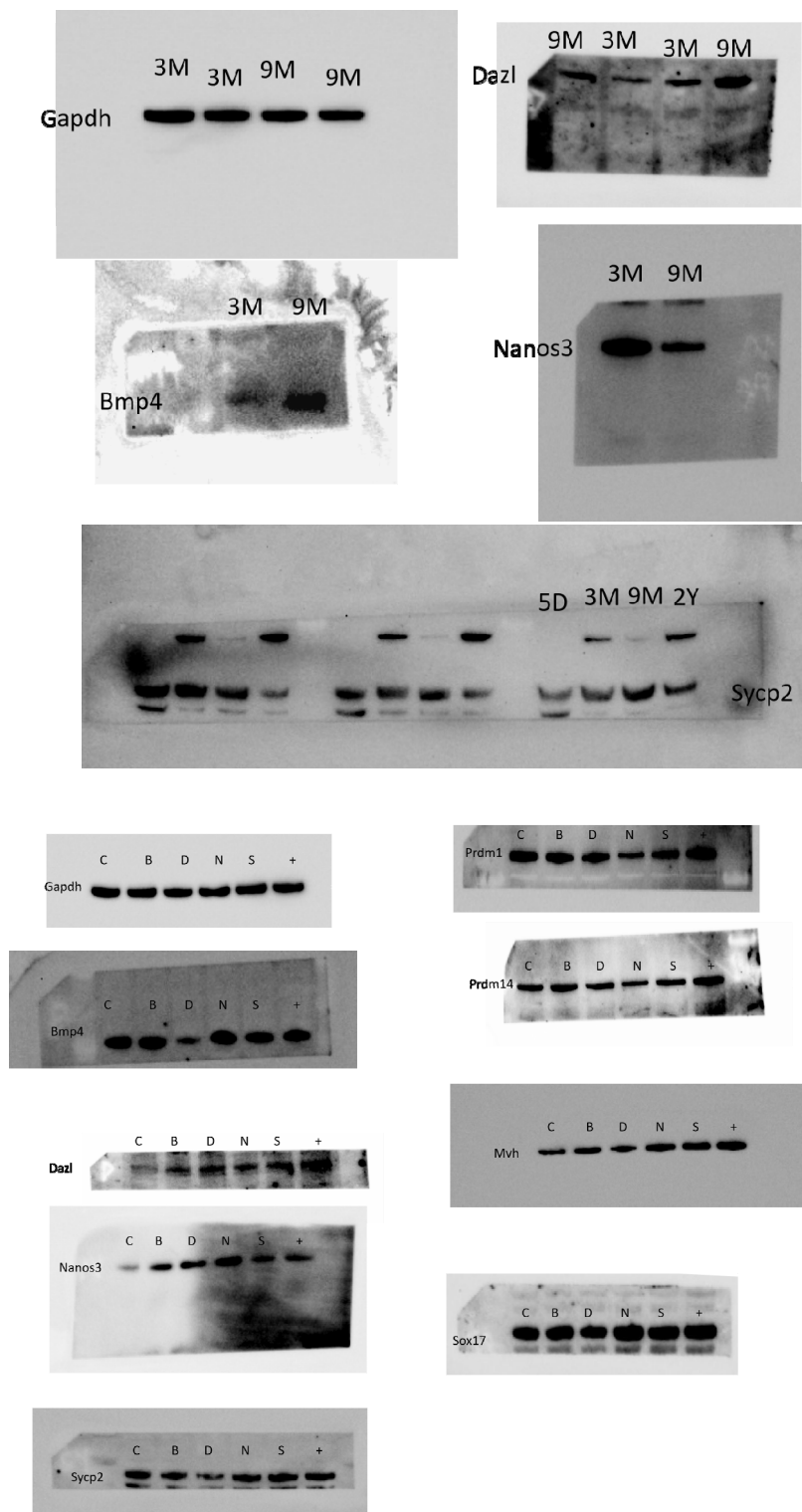

**Figure S2.** The complete western blot images of Figure 2 and 6.

Supplement: Supplementary file 1 [file biology-11-00289-s001.zip › Figure S2.pdf]
